# Prevalence and genomic insights into type III-A CRISPR-Cas system acquisition in global *Staphylococcus argenteus* strains

**DOI:** 10.3389/fcimb.2025.1644286

**Published:** 2025-07-28

**Authors:** Xinhai Chen, Li Xu, Zhijiang Luo, Lihong Wang, Zhenyu Wang, Yang Li, Xinan Jiao, Qiuchun Li

**Affiliations:** ^1^ Jiangsu Key Lab of Zoonosis/Jiangsu Co-Innovation Center for Prevention and Control of Important Animal Infectious Diseases and Zoonoses, Yangzhou University, Yangzhou, China; ^2^ Joint International Research Laboratory of Agriculture and Agri-Product Safety, Yangzhou University, Yangzhou, China; ^3^ Key Laboratory of Prevention and Control of Biological Hazard Factors (Animal Origin) for Agri-food Safety and Quality, Ministry of Agriculture of China, Yangzhou University, Yangzhou, China

**Keywords:** *Staphylococcus argenteus*, CRISPR-Cas, poultry, SCCmec, *IS1272*

## Abstract

**Introduction:**

The CRISPR-Cas system serves as a defense mechanism in bacteria and archaea, protecting them against the invasion of mobile genetic elements. *Staphylococcus argenteus*, a Gram-positive bacterium that diverged from *Staphylococcus aureus*, is characterized by the rare presence of the CRISPR-Cas system in only a few isolates.

**Methods:**

In this study, we analyzed the prevalence of the type III-A CRISPR-Cas system in 368 *S. argenteus* genome sequences from animals, food sources, and humans across 26 countries, available in public database.

**Results:**

Our findings revealed that 44.0% of these strains carry this immune system, with 98.1% of them belonging to the sequence type 2250 (ST2250). Genomic localization analysis indicated that the CRISPR-Cas is closely associated with *SCCmec* (*mecA-ΔmecR1-IS1272-ccrB2-ccrA2*) or Insertion sequence *1272* (IS*1272*) transposase. Further analysis identified a common IS*1272* target inverted repeats (IR) sequence in ST2250 strains, providing insights into why these strains are more likely to acquire the CRISPR-Cas system. CRISPR typing identified 41 sequences types, classifying these strains into two clusters, with Cluster II being the predominant one. Homology analysis of spacers revealed that all the identified 15 spacers exhibited homology to sequences from plasmids, lytic phages, or prophages.

**Conclusion:**

This study suggests that the acquisition of the CRISPR-Cas system in *S. argenteus* enhances its resistance to phage attacks and plasmid invasions in environmental settings, potentially posing significant challenges for clinical treatment of infections caused by these strains and hindering efforts to control their spread in food products using phage-based interventions.

## 1 Introduction

Clustered regularly interspaced short palindromic repeats (CRISPR) and CRISPR-associated (Cas) proteins are considered an adaptive immune system (CRISPR-Cas) distributed in approximately 40% of bacteria and 90% of archaea, protecting them against foreign genetic elements, including phages or plasmids (Barrangou et al., 2007; Kunin et al., 2007; Grissa et al., 2007). A small foreign DNA fragment, ranging mainly from 26 bp to 72 bp, can be inserted into the CRISPR array as a spacer (Grissa et al., 2007). The spacer can be transcribed and processed into mature crRNA, which guides Cas protein complexes to homologous foreign DNA sequences, enabling the digestion of the invading DNA through the activity of Cas nucleases (Hille et al., 2018).

Until now, two classes of CRISPR-Cas systems consist of six types and almost 33 subtypes have been identified (Makarova et al., 2020). The distribution of CRISPR-Cas systems varies across different bacterial species. For example, most *Salmonella* isolates carry the type I-E CRISPR-Cas system, while only a few strains of *S. aureus* and *S. epidermidis* have been reported to contain CRISPR-Cas system (Zhang et al., 2024; Mikkelsen et al., 2023). In staphylococci, the CRISPR-Cas system belongs to type III-A and demonstrates strong activity against phages or plasmids that are targeted by its spacers (Marraffini and Sontheimer, 2008; Li et al., 2021). Besides, the type III-A CRISPR-Cas system doesn’t require a Protospacer Adjacent Motif (PAM) sequence to recognize targeted sequence, distinguishing it from other CRISPR systems like type II CRISPR-Cas9 systems, which relies on a PAM sequence (Pyenson et al., 2017; Gleditzsch et al., 2019). The type III-A CRISPR-Cas system has additional notable characteristics: it can cleave both DNA and RNA targets and induce non-specific immune responses mediated by the production of cyclic oligoadenylate (cOA) by Cas10, which can accumulate nucleases to degrade both foreign and host RNA, leading to an antiviral defense state (Niewoehner et al., 2017; Kazlauskiene et al., 2017). Although the type III-A CRISPR-Cas system show strong immune response against foreign DNAs or RNAs, it is not prevalent in strains of different staphylococci species. Cruz-López et al., found that only 0.83% (6/716) of the analyzed 716 *S. aureus* genomes from GENOMES-NCBI harbored the CRISPR-Cas system (Cruz-López et al., 2021); while our previous study revealed that 2.9% of MRSA isolates in Denmark carried this system (Mikkelsen et al., 2023). A recent study showed that the CRISPR-Cas system existed in all the 40 MDR *S. aureus* isolated from poultry meat in Pakistan (Shabbir et al., 2024). Therefore, it is essential to elucidate the prevalence of type III-A CRISPR-Cas system in staphylococci strains and the genomic characteristics of these strains.

*S. argenteus* was reported as a distinct staphylococcal species diverged from *S. aureus* in 2015, and have been found globally (Tong et al., 2015). It can cause various infections, including bloodstream infection, skin and soft tissue infections, osteomyelitis, and brain abscess like *S. aureus* (Chantratita et al., 2016; Imam et al., 2025; Lee et al., 2024). The major phenotypic difference between the two species is the pigmentation: *S. aureus* typically produces a golden pigment, while *S. argenteus* exhibits a silvery-white appearance (Holt et al., 2011). *rpoB* sequencing is another reliable method to differentiate *S. argenteus* from *S. aureus*, as it reveals species-specific genetic variations in the RNA polymerase β subunit gene (Argudín et al., 2016; Mellmann et al., 2006). Previous studies have analyzed the genomic characteristics of 132 global *S. argenteus* strains from published databases between 2005 and 2008, revealing that ST2550 *S. argenteus* strains exhibit a tendency to carry the type III-A CRISPR-Cas system (Goswami et al., 2021). Since 2008, an increasing number of *S. argenteus* genomic sequences have been submitted to the NCBI GenBank database, and the sources of these strains have expanded. This study further analyzed the prevalence, genetic characteristics, genomic location, and spacer content of the CRISPR-Cas system in 368 *S. argenteus* isolates from across the globe.

## 2 Materials and methods

### 2.1 Data collection of *S. argenteus* strains

A total of 370 genome sequences of *S. argenteus* were obtained from the NCBI GenBank database as of December 31, 2024. Since the *S. argenteus* strains SH3 and DSM 28299 were sequenced twice, 368 unique *S. argenteus* strains and their genomic sequences were included in the analysis for this study. Strain information, including the name, assembly name, accession number, submission data, bioproject number, host, and country of origin, was collected from the database. Additional information was corrected or added based on published papers for the respective strains. The complete information for all 368 strains is provided in the **Supplementary Table 1**.

### 2.2 MLST and *SCCmec* analysis

Multi-locus sequence typing of all *S. argenteus* strains was performed using the PubMLST platform (https://pubmlst.org/organisms/staphylococcus-aureus) designed for *S. aureus*. Although the MLST sequence types (STs) of some strains were known when the genome sequences were submitted to the NCBI GenBank database, all submitted STs were subsequently verified using the PubMLST platform. To identify the presence of *mecA* or *mecC* in the chromosome of *S. argenteus*, we used the SCCmecFinder 1.2 platform (https://cge.food.dtu.dk/services/SCCmecFinder/), which also provides information on the type of *SCCmec* elements and the presence of IS*1272* in the chromosome.

### 2.3 CRISPR-Cas identification

The presence of type III-A CRISPR-Cas system in *S. argenteus* is determined by the *cas* genes (*cas1*, *cas2*, *cas10*, *csm2*, *csm3*, *csm4*, *csm5*, *csm6*, *cas6*) and CRISPR arrays (CRISPR1 and CRISPR2). To assess whether this system is present in *S. argenteus* strains, the CRISPRCasFinder platform (https://crisprcas.i2bc.paris-saclay.fr/CrisprCasFinder) were used to analyze complete genome sequences or assembled contigs. The size of flanking regions for each analyzed CRISPR arrays were set to 100bp, and the repeat length threshold was defined with a minimum of 23 and a maximum of 55. According to the characteristics of CRISPR arrays upstream and downstream of the *cas* gene clusters (Li et al., 2018), small CRISPR-like elements were excluded from the analysis. All the repeats identified in the type III-A CRISPR-Cas system of *S. argenteus* share over 90% homology with the sequences: GATCGATACCCACCCCGAAGAAAAGGGGACGAGAAC. The spacers were extracted from the CRISPR arrays and designed as previously reported in *S. aureus* (Li et al., 2016). All the 15 identified spacers have been previously documented in *S. aureus* (Li et al., 2016).

### 2.4 CRISPR-Cas typing and phylogenic analysis

After extracting the CRISPR arrays from each strain, the spacer arrangements of CRISPR1 and CRISPR2 was analyzed using the spacer names as previously described (Li et al., 2016). Each unique arrangement of spacers in CRISPR1 were designated as “SgCTA + NO.” to represent the CRISPR1 type, while “SgCTB + NO.” was used for the CRISPR2 type. The combination of CRISPR1 and CRISPR2 types was represented as “SgCT + NO.” to indicate the overall CRISPR type for each strain. To perform the genomic analysis of *S. argenteus* strains using CRISPR type, a binary file was constructed based on the presence or absence of spacers. The presence of a spacer was represented by “1”, while the absence was denoted by “0” in the binary file. A phylogenetic tree based on the CRISPR types was then generated using Bionumericus 7.5 software (Applied Maths, Belgium).

### 2.5 Homology analysis of spacers

Previous studies have demonstrated that obtaining homologous sequences of spacers using BLSATn in the NCBI GenBank database is challenging (Li et al., 2016). The advancement of genome sequencing technology and the expansion of phage and plasmid sequences in various databases have significantly improved homology analysis. In this study, the CRISPRTarget (http://crispr.otago.ac.nz/CRISPRTarget/) was used to analyze the homologous sequences of each spacer, employing the GenBank-Phage, RefSeq-Plasmid, and ACLAME databases to represent phage, plasmid, and mobile genetic elements, respectively. The cutoff score was set at 20.

### 2.6 Location of CRISPR-Cas system in chromosome

The CRISPR-Cas system is not present in all the *Staphylococcus* strains; it is considered the genetic elements frequently located within the *SCCmec* region in *S. aureus* (Mikkelsen et al., 2023). Interestingly, most CRISPR-Cas-positive *S. argenteus* strains are methicillin-sensitive. To investigate it further, the contigs or sequences containing the CRISPR-Cas system from these *S. argenteus* strains were extracted and analyzed for the upstream and downstream gene clusters using the SnapGene software (Dotimatics, Boston, USA). Additionally, we searched for IS*1272*-targeted inverted repeats (IR) in the sequences surrounding the CRISPR-Cas system as previously described (Wan et al., 2017).

## 3 Results

### 3.1 Global distribution of *S*. *argenteus* published in the database

**Figure 1:**
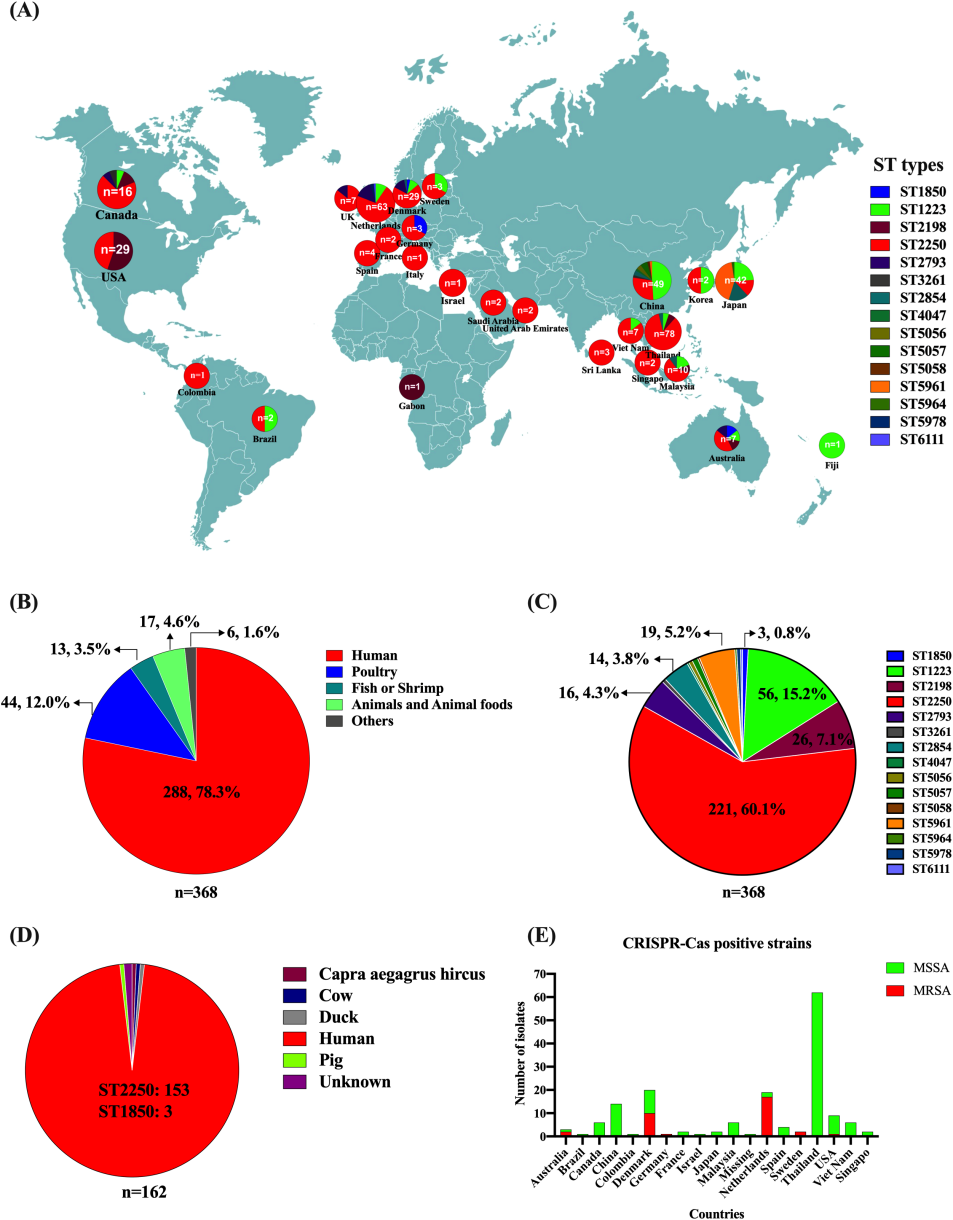
Distribution, Sources, and Characteristics of Global *S. argenteus* isolates. **(A)** Geographic locations and MLST types of *S. argenteus* isolates. **(B)** Number of *S. argenteus* isolates obtained from different hosts. **(C)**. Number of *S. argenteus* with different MLST types. **(D)** Distribution of CRISPR-Cas-positive *S. argenteus* isolates from different hosts. **(E)** Distribution of methicillin-sensitive *S. argenteus* (MSSA) and methicillin-sensitive *S. argenteus* (MRSA) isolates carrying the CRISPR-Cas system across different countries.

Since *S*. *argenteus* was classified as a separate species distinct from *S. aureus* in 2015, the number of *S*. *argenteus* isolates has reached 368 based on the NCBI GenBank database. From 2015 to 2018, 132 publicly available sequences were recorded, while since 2019, more than 230 bacterial genomic sequences have been submitted to the database (**Supplementary Table 1**). The 368 isolates were collected from 26 countries, including Thailand (21.2%, 78), Netherlands (17.1%, 63), China (13.3%, 49), Japan (11.4%, 42), Denmark (7.9%, 29), USA (7.3%, 27), Canada (4.3%, 16), Malaysia (2.7%, 10) and other countries (<10 isolates), such as Australia, Brazil, Colombia, Fiji, France, Gabon, Germany, Israel, Italy, Samoa, Saudi Arabia, Singapore, South Korea, Sri Lanka, Sweden, United Arab Emirates, United Kingdom, and Viet Nam (**Figure 1A**). The geographic distribution of these isolates indicates that *S. argenteus* is now found in Asia, Europe, North America, South America, Oceania, and Africa (**Figure 1A**). The majority of these isolates are sourced from humans (78.3%, 288), including patients and healthy people; however, animals and food products also serve as reservoirs, including poultry (12.0%, 44), fish and shrimp (3.5%, 13), and other livestock (**Figure 1B**). Some isolates have been obtained from vegetables and environmental samples, such as *Capra aegagrus hircus*, chilled water in slaughterhouse, and surface swabs in dental clinics. These findings suggest that *S. argenteus* may be involved in cross-contamination or transmission between foods, animals, and humans.

### 3.2 MLST analysis of *S*. *argenteus*

For the isolates available prior to 2019, the reported MLST types of *S. argenteus* include ST1223, ST1850, ST2198, ST2250, ST2793, ST2854, and ST3261. Since 2019, however, several new sequence types have emerged and increased in number, such as ST5961, ST4067, ST5056, ST5057, ST5058, ST5978, ST5964, and ST6111 (**Figure 1C**; **Supplementary Table 1**). The most prevalent sequence type is ST2250 (60.1%, 221), followed by ST1223 (15.2%, 56), ST2198 (7.1%, 26), ST5961 (5.2%, 19), ST2793 (4.3%, 16), and ST2854 (3.8%, 14). Although the strain MSHR1132 was considered the first *S. argenteus* stain isolated from an indigenous woman with necrotizing fasciitis in 2006, its sequence type is ST1850 (Holt et al., 2011; Li et al., 2016), which has only been reported in three strains (**Figure 1C**; **Supplementary Table 1**). The predominant sequence type remains ST2250, accounting for 60.1% of the global isolates (**Figure 1C**).

### 3.3 CRISPR-Cas positive *S. argenteus*

Staphylococcal species possess a type III-A CRISPR-Cas system located on the chromosome, though not all isolates carry this system. The type III-A CRISPR-Cas system in *S. epidermidis* and *S. aureus* has been shown to protect bacteria against phage attacks and plasmid invasion. The presence of the CRISPR-Cas system is more common in *Staphylococcal* isolates with specific MLST sequence types; for example, 50% of *S. aureus* ST630 isolates carry the system. In *S. argenteus*, the CRISPR-Cas system is present in all ST2250 isolates, except for three ST1850 isolates that also carry the system. It is not found in any other ST isolates (**Figure 1D**). The three ST1850 isolates were collected from human infections in Australia, Germany, and Denmark (**Figure 1D**). Among the 221 ST2250 isolates, 159 (71.9%) carry the CRISPR-Cas system, while 62 are negative of the system. The 159 CRISPR-Cas-positive ST2250 isolates were collected from 17 out of 24 countries (**Figure 1E**). The three ST1850 isolates were methicillin-resistant due to the presence of *mecA* gene. In contrast among the 159 CRISPR-Cas-positive ST2250 isolates, only 30 (18.9%) were MRSA, with the majority of these strains collected from Netherlands and Denmark (**Figure 1E**).

### 3.4 CRISPR types of *S. argenteus*

**Figure 2:**
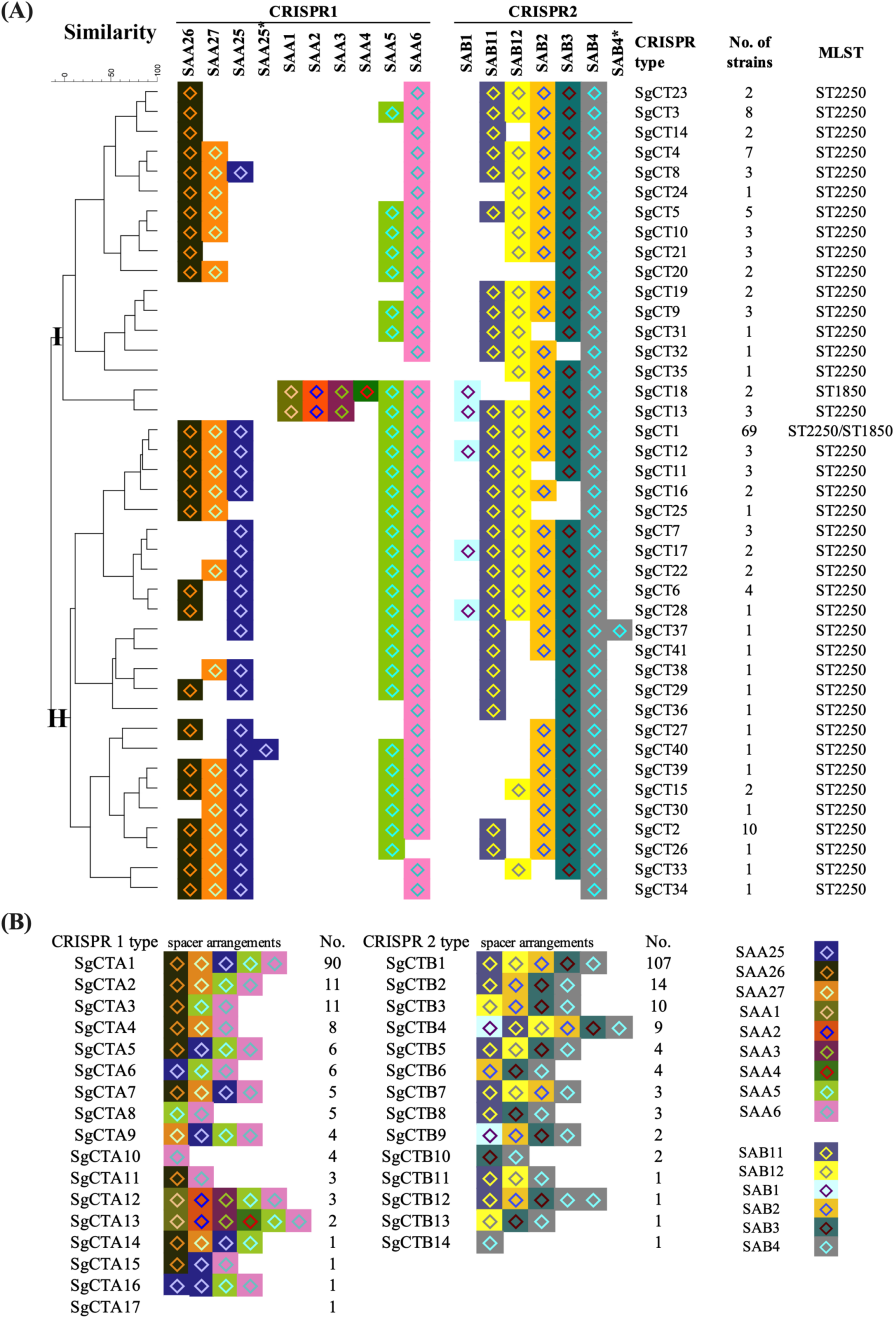
CRISPR typing of CRISPR-Cas-positive *S. argenteus* isolates. **(A)** Phylogenetic tree of CRISPR-Cas-positive *S. argenteus* isolates based on 41 distinct CRISPR types. The spacer arrangements in the CRISPR 1 and CRISPR 2 loci were used to define CRISPR types. The number of strains corresponding to each CRISPR type and their associated MLST types are indicated in the right columns. **(B)**. Distribution of strains with CRISPR 1 or CRISPR 2 types, with spacer arrangements shown for each type.

Although only 15 spacers were identified in all the CRISPR-Cas-positive isolates, 41 *S. argenteus* CRISPR types (SgCTs) were detected according to the arrangement of spacers (**Figure 2A**). As shown in **Figure 2A**, the most prevalent CRISPR type was SgCT1, accounting for 42.6% (69) of the 162 CRISPR-Cas-positive isolates. The spacers arrangement of SgCT1 is SAA26-SAA27-SAA25-SAA5-SAA6 (SgCTA1) for CRISPR1 locus and SAAB11-SAB12-SAB2-SAB3-SAB4 (SgCTB1) for CRISPR2 locus (**Figures 2A, B**). Analysis of the CRISPR types for each CRISPR locus showed that 90 isolates share the SgCTA1 for CRISPR1 locus, and 107 isolates share the SgCTB1 for CRISPR2 locus (**Figure 2B**). Ten isolates shared the SgCT2, which has SAB12 deleted in the CRISPR2 locus compared to that of SgCT1 (**Figure 2A**). Eight isolates belong to the SgCT3, which has SAA27 and SAA25 deleted in the CRISPR1 locus compared to that of SgCT1 (**Figure 2A**). Deletion or addition of spacers led to the emergence of new CRISPR types in *S. argenteus*. Cluster analysis grouped the 41 SgCTs into two clusters, Cluster I and Cluster II (**Figure 2A**). Seventeen SgCTs, covering 49 (30.2%) strains, belong to Cluster I, while 24 SgCTs, covering 113 (69.8%) strains, belong to Cluster II (**Figure 2A**). SgCT1 and SgCT2, located in Cluster II, account for 48.8% of the strains, indicating that Cluster II is the predominant group among CRISPR-Cas-positive *S. argenteus* strains (**Figure 2A**).

Among the nine spacers in the CRISPR1 locus, the most prevalent spacer is SAA6 (160), followed by SAA5 (150), SAA26 (136), SAA25 (125), and SAA27 (119) (**Figure 2B**). However, the spacers SAA1, SAA2, and SAA3 in CRISPR1 locus were found only in three ST2250 and two ST1850 isolates; while the spacer SAA4 was detected in the two ST1850 isolates (**Figure 2A**). Among the six spacers in the CRISPR2 locus, SAB4 is present in all 162 CRISPR-Cas-positive isolates, followed by SAB3 (157), SAB2 (150), SAB11 (141), and SAB12 (115). The spacer SAB1 was found only in two ST1850 and nine ST2250 isolates (**Figure 2A**).

**Figure 3:**
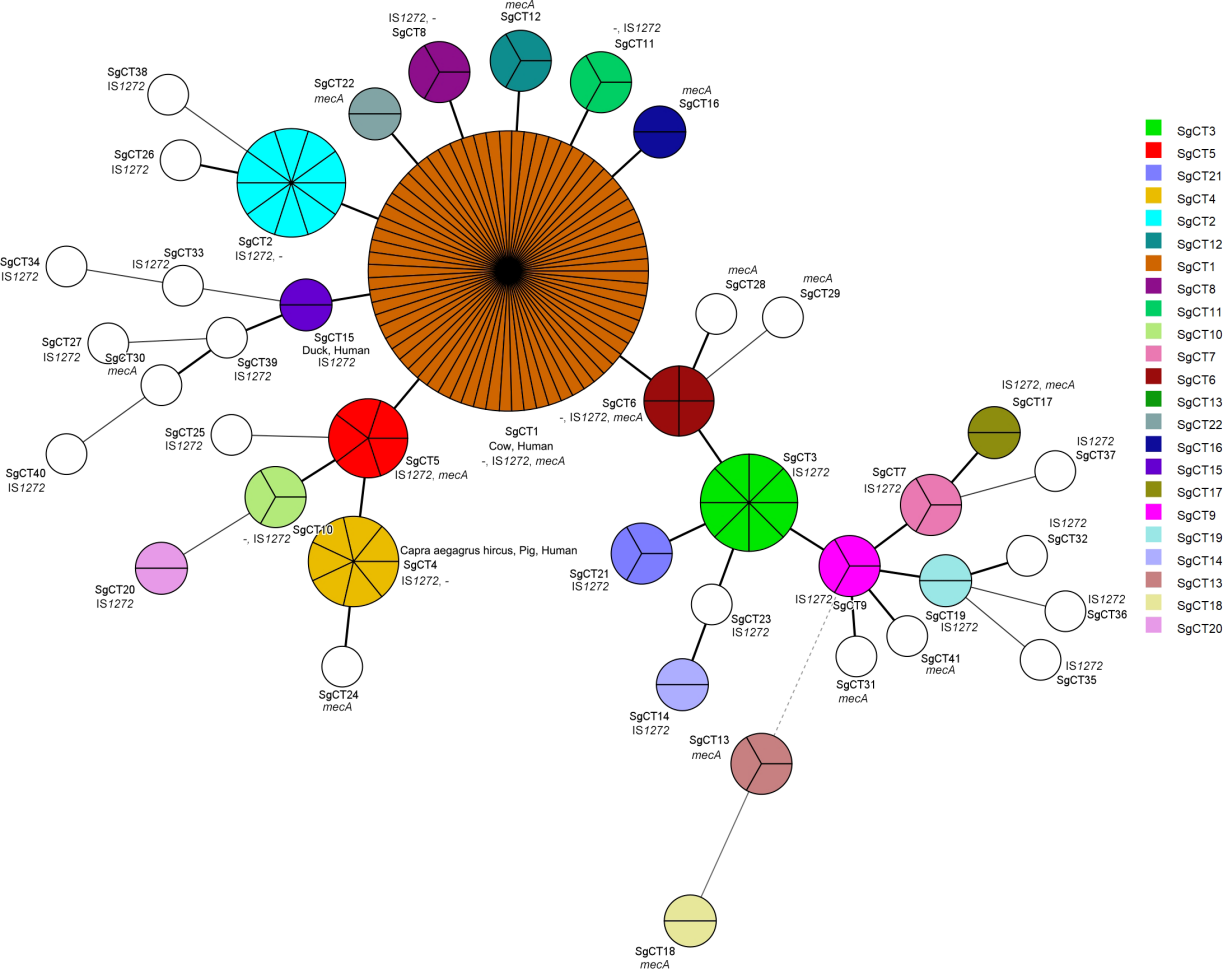
Genetic relationship and characteristics of CRISPR-Cas-positive *S. argenteus* isolates. The Minimum spanning tree of CRISPR-Cas-positive *S. argenteus* isolates were constructed using BioNumerics 7.5 software based on CRISPR types. Each circle represents the strains sharing a single CRISPR type. The presence of *mecA* and IS*1272* is indicated in each circle, while “-” indicates strains lacking both *mecA* and IS*1272*.

The minimum spanning tree graph (MST) graph, generated using the BioNumerics v7.5 advanced cluster analysis tool, revealed the relationships between each SgCT (**Figure 3**). Interestingly, all 41 CgSTs include isolates with either IS*1272* or *mecA* in their genomes; while seven SgCTs (SgCT1, 2, 4, 6, 8, 10, and 11) contain 14 isolates that lack both IS*1272* and *mecA*, indicating that 91.3% (146/160) of the isolates harbor IS*1272* in the chromosome (**Figure 3**; **Supplementary Table S3**). Among the methicillin-resistant *S. argenteus* isolates, two types of SCC*mec* were identified: type_IVa(2B) and type_IVc(2B) (**Supplementary Table S3**).

### 3.5 Homology analysis of CRISPR spacers

Although no novel spacers were identified in *S. argenteus*, the homology analysis of these spacers was significantly enhanced due to the increased availability of genomic sequences of phages, plasmids, prophages in public databases. Consequently, we conducted homology analysis of these spacers in the CRISPRTarget platform (**Table 1**). Among the 15 spacers, 13 show similarities to phage sequences, with12 spacers specifically homologous to *Staphylococcus* phage sequences. Additionally, four spacers exhibited homology to sequence in plasmids, three of which were identified as *S. aureus* plasmids. In details, the spacer SAA2 and SAA25 showed homology to different sequences in the same *Staphylococcus* phage qdsa001, a lytic phage isolated from urban sewage and used to inactivate *S. aureus* in ready-to-eat milk in China. The spacer SAA6 showed 100% homology to sequences located in a gene encoding a DUF1270 family protein, which is present in both the *S. aureus* plasmid pSALNBL118 and the lytic phage vB_SauS-SAP27. Although SAA3 showed similarity to sequences in both the *Phocaeicola salanitronis* plasmid pBACSA01 and the proPHAGE_Entero_phiFL1A, its homology rate is much lower compared to other spacers. The spacers SAA4, SAA26, SAA27, and SAB1 exhibited homology to sequences in the lytic phages SA11, SAP-2, GRCS, and vB_Sau-RP15, respectively. Meanwhile, SAA1, SAA5, SAB2, and SAB3 showed homology to sequences in the prophages PT1028, B236, phiMR25, and StauST398-5, respectively. Additionally, SAB4 and SAB12 were homologous to sequences located in a plasmid of *S. aureus* strain AR_0471 and the plasmid pWBG731, respectively.

**Table 1:** Homology analysis results of spacers from *S. argenteus*.

Spacers	Sequences	Homology to plasmid sequences	Homology to phage sequences
SAA1	TCTACTAAAAAGTTATATGTTTCAACAATTTCGTCA		28/36, *Staphylococcus* phage PT1028
SAA2	TGGTTTAAGTTTGTCATTATAATCAATCCTTTTTCTT		34/37, *Staphylococcus* phage qdsa001
SAA3	TGATTAAAACGGTTTGCTTTATTTGCATTTAAAATAG	29/37, *Phocaeicola salanitronis* DSM 18170 plasmid pBACSA01	27/37, proPHAGE_Entero_phiFL1A
SAA4	GTTTTTCATAGTTAATCAATCCCTTTTCTTTTTT		31/34, *Staphylococcus* phage SA11
SAA5	TTAAATCTTTGATTGCTCTTAGCTCTAGTTATGTAT		33/36, *Staphylococcus* phage B236
SAA6	CACGCTGTAGTGAAGTATAGAAACGGCATGAGTACAAT	38/38, *Staphylococcus aureus* plasmid pSALNBL118	38/38, *Staphylococcus* phage vB_SauS-SAP27
SAA25	CAATATCTTGTACATGGTTATCAAAGAAAGTTACGATC		33/38, *Staphylococcus* phage qdsa001
SAA26	GAGCATTATTTACAAACAAAGAATCAAAATTCGG		33/34, *Staphylococcus* phage SAP-2
SAA27	TTAATTGCATTATCAAATGTATATGCTGGATTCCA		33/35, *Staphylococcus* phage GRCS
SAB1	TTTTACTGTGTTTTTCATAATTAATCAATCCTTT		34/34, *Staphylococcus* phage vB_Sau-RP15
SAB2	TGCCCACTTAATTAATTCATCTAGTCTCATTTCTT		34/34, *Staphylococcus* phage phiMR25
SAB3	CATCAACTGACTTTTTAACTGTTTTAGTGAATTCGTC		37/37, *Staphylococcus* phage StauST398-5
SAB4	TTAAAGATCTCAACAATAGCGTCCCATATTTTCTG	34/35, *Staphylococcus aureus* strain AR_0471 plasmid unnamed1	
SAB11	CTATAATAGTTACTGCTTTTGTAACCGTCCATAT		32/34, *Staphylococcus* phage vB_SauM-V1SA20
SAB12	AAATGCTTATCCATTCTAATCATATTTTCAATTTGTTTA	33/39, *Staphylococcus aureus* strain WBG10514 plasmid pWBG731	

### 3.6 Genetic location of CRISPR-Cas system in *S. argenteus*

**Figure 4:**
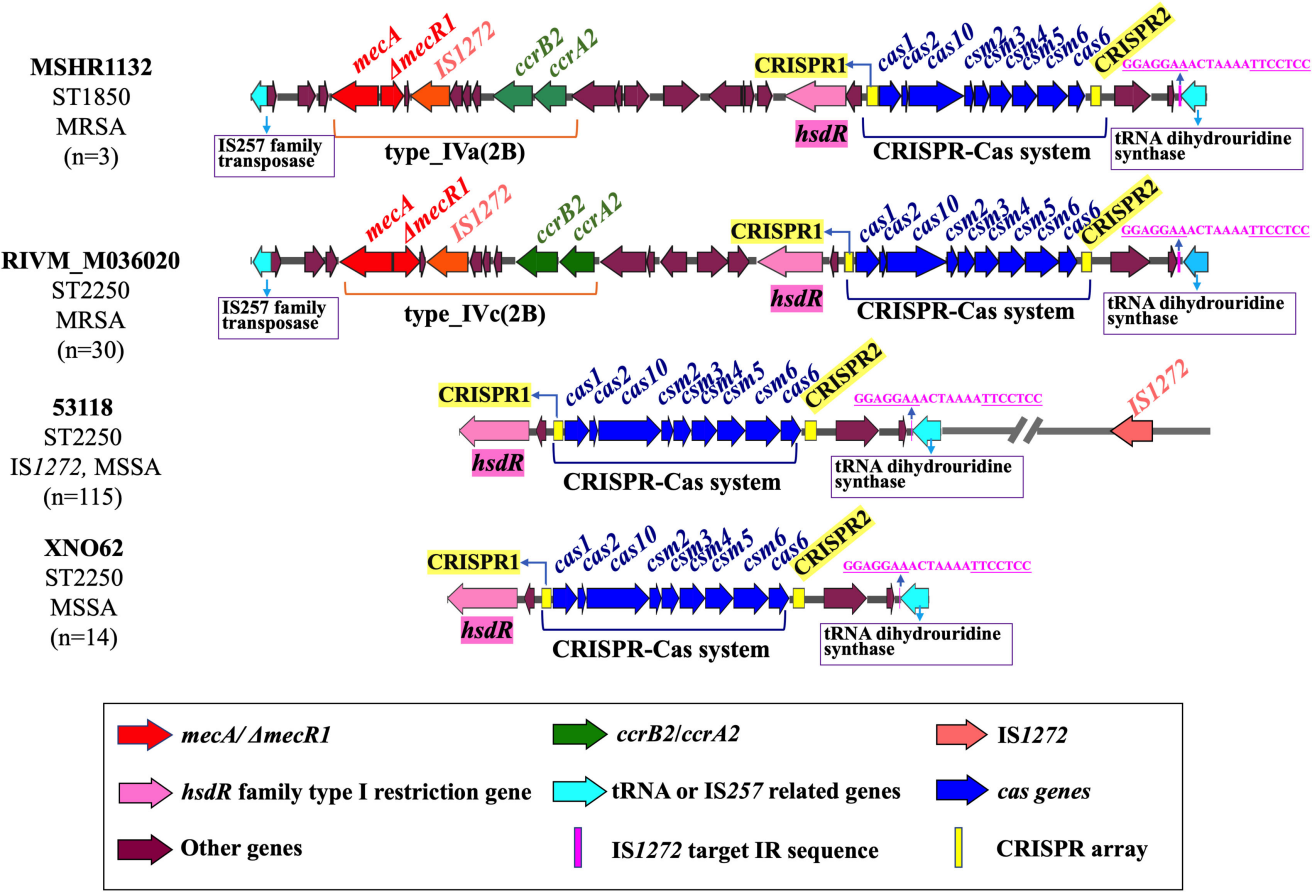
Genetic location of CRISPR-Cas system in the *S. argenteus* chromosome. The arrangement and chromosomal location of the CRISPR-Cas system are illustrated in MSSA and MRSA strains with two distinct MLST types. The conserved IS*1272* target IR sequences are also highlighted in these strains.

To analyze the genomic location of the type III-A CRISPR-Cas system in *S. argenteus*, the contigs containing the CRISPR-Cas system were collected for identification of the sequences upstream of CRISPR1 array and downstream of CRISPR2 array. According to the difference of MLST sequence types, the presence of *mecA* or IS*1272*, we determined the location sites of the CRISPR-Cas system for four different types of strains (**Figure 4**). The three ST1850 strains were identified as methicillin-resistant *S. argenteus*, with the CRISPR-Cas system located downstream of *hsdR* and adjacent to the *SCCmec* type Iva(2B) cassette (**Figure 4**). Among the 30 ST2250 methicillin-resistant *S. argenteus* strains, the CRISPR-Cas system exhibited a similar genomic location to that of the ST1850 strains, but these strains carried two types of *SCCmec* cassettes: type IVa(2B) and type IVc(2B) (**Figure 4**). Among the 129 CRISPR-Cas-positive ST2250 methicillin-sensitive *S. argenteus* strains, 115 (89.1%) strains carry IS*1272* elements in their chromosomes (**Figure 4**). Interestingly, at the downstream location of the CRISPR2 locus, a common IS*1272* target inverted repeat (IR) site, GGAGGAAACTAAAATTCCTCC, was identified, which is located near the gene encoding tRNA dihydrouridine synthase (**Figure 4**). Additionally, all ST2250 strains carry the target IR site, suggesting that ST2250 strains are more likely to acquire the CRISPR-Cas system compared to strains of other sequence types. The presence of IS*1272* within the *SCCmec* cassette further explains why the acquisition of the CRISPR-Cas system is closely associated with the presence of *mecA*.

## 4 Discussion

As an adaptive immune system of bacteria, the CRISPR-Cas system provides protection against foreign phages and plasmids. However, this system is not widespread among staphylococci species, as only certain isolates with specific characteristics tend to carry it (Rossi et al., 2017). For instance, more than 50% of ST630 MRSA strains have been found to carry the CRISPR-Cas system (Mikkelsen et al., 2023). *S. argenteus* is a recently identified species capable of causing human infections and is frequently detected in food, particularly in poultry products (Wakabayashi et al., 2022; Li et al., 2019). *S. argenteus* was first identified in Australia and has since been increasingly reported worldwide, particularly in tropical regions (McDonald et al., 2006). In Thailand, it has been associated with community-acquired invasive infections since 2006 (Thaipadungpanit et al., 2015). As a result, numerous strains have been collected and sequenced for comparative analysis with *S. aureus* (Chantratita et al., 2016). Additionally, an increasing number of *S. argenteus*-related cases have been reported in other countries, including the Netherlands, Japan, China, etc (Aung et al., 2025; Chen et al., 2023; Bank et al., 2021). To date, 15 STs have been identified in *S. argenteus*, with ST2250 (60.1%) and ST1223 (15.2%) being the most prevalent STs globally. In Japan, ST2250 took up 49% of clinical *S. argenteus* isolates collected from 2020 to 2023 (Aung et al., 2025). ST2250 has been reported as the predominant ST in both food sources and clinical isolates from various regions, including Indonesia (Supriadi et al., 2024), Hong Kong (China) (Chen et al., 2023), Guangdong (China) (Rong et al., 2023), Myanmar (Kyaw et al., 2023), North America (Eshaghi et al., 2021), Hokkaido (Japan) (Aung et al., 2021). In our study, 71.9% of the ST2250 strains were found to carry the CRISPR-Cas system, suggesting that these strains may have enhanced resistance against phage infections. This is supported by the previous findings that the CRISPR-Cas system in *S. aureus* and *S. epidermidis* are functionally active in providing immunity against phage and plasmid infections (Marraffini and Sontheimer, 2008; Li et al., 2021). This may also explain the higher prevalence of CRISPR-Cas-positive *S. argenteus* in Thailand compared to other countries, as nearly 84.6% (66/78) of the clinical isolates belonged to ST2250. Although the genetic location of the CRISPR-Cas system in *S. aureus* and *S. argenteus* has been reported to be closely associated with *SCCmec* (Mikkelsen et al., 2023; Goswami et al., 2021), the characteristics of the specific insertion sites for the CRISPR-Cas system have not yet been analyzed in detail. Here, we found that the CRIPSR-Cas system in *S. argenteus* is most closely related to the IS*1272* transposase, which is also detected in *SCCmec* elements of methicillin-resistance *S. argenteus*, suggesting that IS*1272* may play a role in the acquisition of the CRISPR-Cas system in *S. argenteus.* Additionally, in all the CRISPR-Cas-positive *S. argenteus*, a conserved recognition site for IS*1272* inverted repeat (IR) elements is consistently observed at the right end of the CRISPR2 locus. CRISPR typing, developed as a molecular typing method, has been widely used to reveal the genetic difference and evolutionary relationships among different bacterial isolates, showing strong correspondence with cgMLST (core genome multilocus sequence typing) and cgSNP (core genome single nucleotide polymorphism) typing methods (Li et al., 2016; Yassine et al., 2022). In this study, CRISPR typing classified the CRISPR-Cas-positive *S. argenteus* isolates into two clusters, and the Cluster II emerging as the predominant group among these strains.

The spacers within CRISPR array play a crucial role in providing adaptive immunity against foreign genetic elements. The 15 spacers identified in *S. argenteus* have also been reported in *S. aureus*, suggesting that both species encounter and survive in environments with similar phages and plasmids. However, obtaining the homologous sequences for these spacers has been challenging due to the limited availability of comprehensive databases in earlier studies (Mikkelsen et al., 2023; Li et al., 2019). Here, the homologous sequences for all 15 spacers were successfully identified using multiple databases, providing new insights into the targets of these spacers and their role in adaptive immunity. Among the identified spacers, four showed homology to sequences in plasmids. pWBG731 is a multidrug resistance plasmid frequently found in community-associated methicillin-resistant *S. aureus* (CA-MRSA) (Yui Eto et al., 2019). The plasmid carries genes conferring resistance to mupirocin, trimethoprim, cadmium, and penicillin, as well as mobile genetic elements related to the horizontal dissemination of multidrug resistance in CA-MRSA (Yui et al., 2019). pSALNBL118 is a phage like plasmid originating from *S. aureus* strain B3–4A, isolated from beef liver (Karki et al., 2020). The plasmid is thought to play a significant role in horizontal gene transfer (Goerke et al., 2009) and transmission of virulence factors.

Among the 13 spacers with homology to phage sequences, seven correspond to lytic phages, while six were associated with lysogenic phages. SA11 is a lytic phage isolated from a wastewater treatment facility in Gwa-Chon, South Korea (Kim and Myung, 2012). It has been successfully used in combination with antibiotics to effectively inhibit the growth of antibiotic-resistant *S. aureus* under simulated intestinal conditions (Zhou et al., 2023). SAP-2 is a podoviridae lytic bacteriophage that encodes a cell-wall degrading enzyme, SAL-2, which can disrupt biofilm formation of *S. aureus*, including MRSA (Son et al., 2010). GRCS is a podoviridae lytic phage isolated from sewage in India, and it showed more efficient in treating both diabetic and non-diabetic septicemic mice than oxacillin antibiotic alone (Plumet et al., 2022). The vB_Sau-RP15 phage was isolated from raw milk and developed as a promising agent against *S. aureus* contamination in pasteurized milk (Imklin et al., 2023). Phage vB_SauM-V1SA20, isolated from wastewater, exhibits a broad host activity against *S. aureus*, including CC80 strains (Kolenda et al., 2022). Phage vB_SauS-SAP27 (ϕSAP27) is a Siphovirdiae phage that infects *S. aureus* and was isolated from sewage (Park et al., 2021).

The homology of spacers to temperate phages was not given much consideration during the CRISPR-Cas system analysis. Among the six temperate phages, phage B236 has been identified as an *eta* (Exfoliative toxin A, ETA) phage, contributing to the toxic phenotype of the *S. aureus* SA236 strain (Botka et al., 2015). This kind of phages are able to mediate the transfer of *eta* gene to prophage-free *S. aureus* strains. ETA is potentially a major toxin responsible for staphylococcal skin blistering infections (Botka et al., 2015). Phage phiMR25 was a lysogenic phage isolated from an MRSA strain MR25 by mitomycin C induction (Hoshiba et al., 2010). Although it is a lysogenic phage, is showed a broad host range and can protect mice against *S. aureus* infection (Hoshiba et al., 2010). The prophage StauST398–5 was specifically identified in non-LA CC398 isolates, where it protects bacteria from horizontal genetic transfer to its host and carries genes related to bacterial virulence and adaptation (van der Mee-Marquet et al., 2013).

## 5 Conclusion

In this study, the presence of the type III-A CRISPR-Cas system in 368 *S. argenteus* strains obtained from public database were analyzed to reveal the genetic characteristics of CRISPR-Cas-positive strains. The CRISPR-Cas system is present in 44.0% (162) of *S. argenteus* strains, but only in ST2250 and ST1850 strains. Notably, ST2250 strains, which are the predominant sequence type of *S. argenteus*, show that 71.9% of these strains carry the CRISPR-Cas system. Additionally, the presence of IS*1272* and its target IR site is likely the reason for the acquisition of the CRISPR-Cas system in ST2250 strains. Homology analysis confirmed that all 15 identified spacers in the CRISPR array showed homology to sequences in plasmids, phages, or prophages, indicating that the acquisition of the CRISPR-Cas system may provide protection against phage attacks and plasmid invasion. These findings highlight the potential role of the CRISPR-Cas system in enhancing the adaptive immunity of *S. argenteus* in environments rich in mobile genetic elements.

## Data Availability

The original contributions presented in the study are included in the article/**Supplementary Material**. Further inquiries can be directed to the corresponding authors.
